# The skill of tracheal intubation with rigid scopes – a randomised controlled trial comparing learning curves in 740 intubations

**DOI:** 10.1186/s12871-020-01181-w

**Published:** 2020-10-16

**Authors:** Lorenz Theiler, Robert Greif, Lukas Bütikofer, Kristopher Arheart, Maren Kleine-Brueggeney

**Affiliations:** 1 Department of Anaesthesia, Cantonal Hospital Aarau, Aarau, Switzerland; 2 Department of Anaesthesiology and Pain Medicine, Inselspital, Bern University Hospital, University of Bern, 3010 Bern, Switzerland; 3 School of Medicine, Sigmund Freud University Vienna, Vienna, Austria; 4 CTU Bern, University of Bern, Bern, Switzerland; 5 Department of Public Health Sciences, University of Miami Miller School of Medicine, Miami, Florida, USA; 6 Department of Anaesthesia, University Children’s Hospital Zurich – Eleonore Foundation and University of Zurich, Steinwiesstrasse 75, 8032 Zurich, Switzerland

**Keywords:** Learning curves, Procedural skills, Rigid scopes, Difficult airway management, Tracheal intubation

## Abstract

**Background:**

Rigid scopes are successfully used for management of difficult airways, but learning curves have not been established.

**Methods:**

This randomised controlled trial was performed at the University Hospital Bern in Switzerland to establish learning curves for the rigid scopes Bonfils and SensaScope and to assess their performance. Fifteen consultant anaesthetists and 15 anaesthesia registrars performed a total of 740 intubations (10 to 20 intubations with each device per physician) in adult patients without predictors of a difficult airway under general anaesthesia. According to randomisation, physicians intubated the patient’s trachea with either the Bonfils or the SensaScope. A maximum of three intubation attempts was allowed. Primary outcome was overall time to successful intubation. Secondary outcome parameters included first attempt success, first attempt success within 60 s, failures and adverse events.

**Results:**

A clear learning effect was demonstrated: Over 20 trials, intubations became 2.5-times quicker and first attempt intubation success probability increased by 21–28 percentage points. Fourteen and 20 trials were needed with the Bonfils and the SensaScope, respectively, to reach a 90% first attempt success probability. Intubation times were 23% longer (geometric mean ratio 1.23, 95% confidence interval 1.12–1.36, *p* < 0.001) and first attempt success was less likely (odds ratio 0.64, 95% confidence interval 0.45–0.92, *p* = 0.016) with the SensaScope. Consultants showed a tendency for a better first attempt success compared to registrars. Overall, 23 intubations (10 Bonfils, 13 SensaScope) failed. Adverse events were rare and did not differ between devices.

**Conclusions:**

A clear learning effect was demonstrated for both rigid scopes. Fourteen intubations with the Bonfils and 20 intubations with the SensaScope were required to reach a 90% first attempt success probability. Learning of the technique seemed more complex with the SensaScope compared to the Bonfils.

**Trial registration:**

Current Controlled Trials, ISRCTN14429285. Registered 28 September 2011, retrospectively registered.

## Background

Rigid intubation scopes are used for management of predicted and unpredicted difficult airways [1–4]. Compared with flexible optical scopes, rigid scopes provide faster set-up, a more durable lifespan, and an easy cleaning process [5]. They were shown to improve success compared to the Macintosh laryngoscope [4], and oral intubation was faster compared to flexible fibrescopes [6, 7].

Two rigid intubation scopes used for intubation of difficult airways are the Bonfils™ (Karl Storz GmbH, Tuttlingen, Germany) and the SensaScope™ (Acutronic Medical Systems AG, Hirzel, Switzerland). The SensaScope is an S-formed semirigid scope with a steerable, flexible tip [3]. The Bonfils is straight with a rigid curved tip. Both scopes enable oxygen administration over an attached tracheal tube and an adapter. Success rates of over 80% have been described for the Bonfils in a simulated difficult airway setting [4]. Similarly, SensaScope and Bonfils both achieved overall intubation success rates of 88–89% in a randomized controlled trial in patients with a simulated difficult airway [1].

Apart from the quality and design of the used devices and non-technical skills, proficiency with the equipment is the crucial factor for successful airway management. Efficient methods to teach airway management as well as case numbers required to achieve proficiency are subject to discussion. In a clinical environment with an increasing number of techniques and simultaneously decreasing numbers of anaesthesia cases per anaesthetist caused by part time work and decreasing working hours, acquisition of new skills in an efficient manner is becoming more and more important.

Learning processes are often described by learning curves [3, 8–10]. However, studies have used different statistical methods to establish learning curves and it is unclear which method is best. Due to limitations of statistical methodology or due to true differences in learning profiles, studies have provided differing results regarding the numbers required to achieve proficiency with direct laryngoscopy [8, 9, 11], flexible fibreoptic intubation [10], and other intubation tools [3, 9, 11, 12]. Learning curve studies suggested that perhaps as few as 18 intubations may be required to achieve adequate experience to perform flexible fibreoptic intubation in a non-difficult airway [10].

For the SensaScope, no learning curve studies in patients are available, but it has been reported that only 2 uses might be enough to achieve proficiency, suggesting that rigid scopes might be easier to handle than flexible devices [3]. For the Bonfils, studies suggest that around 20 intubations might be needed to achieve proficiency [12, 13]. In contrast, a recent study in manikins suggested superiority of the Bonfils over the SensaScope when used by novices [14]. These differing figures underline the fact that knowledge about learning curves are inconsistent. No study compared the SensaScope and the Bonfils.

The present study used advanced statistical models to establish learning curves of tracheal intubation using the SensaScope and the Bonfils when used by anaesthesia registrars or by anaesthetic consultants. These models could serve as an example for future learning curve studies for other techniques.

## Methods

This randomized controlled trial evaluates the learning curves for tracheal intubation with the rigid scopes SensaScope and Bonfils. It was performed at the University Hospital in Bern, Switzerland, following CONSORT guidelines. It was part of a research project about the two rigid scopes including the presented learning of intubation in patients with normal airways, and an assessment of the performance of the scopes in patients with a simulated difficult airway, which was previously published [1]. Ethical approval was provided by the local ethics committee (Cantonal Ethics Committee, Bern University Hospital, Bern, Switzerland, Chairperson Dr. Christian Seiler; approval number KEK 247/09, 22/02/2010, amendments approved on 31/05/2012 and 25/06/2012). Registration was done retrospectively through Current Controlled Trials (ISRCTN14429285).

Written informed consent was obtained from all participating doctors and all patients gave written consent to use their health-related personal data for research purposes. Two groups of anaesthetists were included: registrars and consultants. Registrars had less than 2 years clinical experience and had experience in direct laryngoscopy, but had not performed more than 10 fibreoptic intubations. Consultants had more than 8 years clinical experience and were proficient in direct laryngoscopy and flexible fibreoptic intubation (> 70 fibreoptic intubations), thus having much more experience than the postulated necessary 60 conventional intubations and 18 fibreoptic intubations to achieve proficiency [8, 10]. None of the participating doctors had experience with the SensaScope or the Bonfils. Anaesthetists fulfilling these criteria, and willing and available to participate were included. Prior to the start of the study participants were instructed regarding both devices in a one-to-one session, which included practice on an intubation manikin (Laerdal® Airway Management Trainer, Stavanger, Norway).

Computer-generated randomization was performed in blocks for each participating doctor. Randomization numbers to use the Bonfils or SensaScope were in sealed opaque envelopes which were opened by the study personnel after induction of anaesthesia, while bag mask ventilation was provided.

Patients of both genders, aged 18–85 years, ASA physical status I to III, undergoing elective surgery under general anaesthesia requiring tracheal intubation were intubated by the participating doctors. Exclusion criteria were risk of aspiration, known difficult mask ventilation and mouth opening < 30 mm.

All patients were prepared and monitored for anaesthesia according to the standard operating procedures of the Bern University Hospital. Anaesthesia was induced with propofol or etomidate, fentanyl with or without remifentanil, and rocuronium or atracurium. Neuromuscular blockade was confirmed by loss of 1 Hz muscle twitching (TOF Watch, Organon, Dublin, Ireland) [1, 15].

According to randomization, either the SensaScope or the Bonfils was used for intubation. A Macintosh laryngoscope was used to create pharyngeal space. No direct laryngoscopy was performed. Both scopes were used as previously described [1, 3, 5]: With the SensaScope, the right hand was used to open the mouth, the left hand handled the Macintosh laryngoscope to elevate the tongue. The SensaScope, connected to a video unit for visualisation, was then advanced in a midline approach. After passage of the glottis the mounted tracheal tube was railroaded over the scope. For the Bonfils, the retromolar approach from the right side of the mouth was used [5], and the tube was advanced into the trachea under visualisation of the glottis. Tube manipulations to facilitate advancement into the trachea were rotation 90° anticlockwise followed by a second rotation 90° anticlockwise if necessary [16]. No supplemental oxygen was administered via the scopes. Tracheal tube size selection was according to gender: Inner diameter 7.0 mm for women and 8.0 mm for men. Presence of end-tidal CO_2_ waveforms confirmed the tracheal tube position [17], which represented the formal end of the study intervention. Anaesthesia was then continued according to the consultant anaesthetist.

The intubation procedure with a device was stopped if one of the following criteria was met: Three unsuccessful intubation attempts, soft tissue trauma, bronchospasm, laryngospasm, failing bag-mask ventilation between intubation attempts, or oesophageal intubation. The patient’s airway was then managed according to the consultant anaesthetist. An attempt was abandoned after a maximum of 120 s [4] or if oxygen saturation fell below 93%. If the tracheal tube was already being advanced after 120 s the intubation attempt was not abandoned as long as oxygen saturation remained above 93%. However, intubation beyond 120 s of the first or second attempt was not rated as a success of this attempt, but only as overall success. Intubation beyond 120 s of the third attempt was counted as overall failure. Bag-mask ventilation was instituted between attempts.

For clarity we are using the following terms: An attempt was defined as the uninterrupted process to intubate a patient’s trachea with the device. A maximum of three attempts with a maximum of 120 s each were allowed per patient. A trial was defined as the use of a device on a specific patient, i.e. a maximum of three attempts to intubate the trachea during the same anaesthesia.

### Data collection and outcome parameters

Data collection was performed by a member of the research group who was not involved in the clinical care of the patient. Standard demographic data like sex, age, height, weight, body mass index, ASA class and Mallampati score were recorded.

Primary outcome parameter was overall time to successful intubation. Intubation time was measured from the moment the face mask was taken away from the face until the tube was placed and cuffed in the trachea. In case of several intubation attempts the overall intubation time to successful intubation of the trial was calculated as 120 s for each failed attempt plus the time needed for the successful attempt.

Secondary outcome parameters included first attempt success. Additionally, first attempt success within 60 s was analysed. Intubation failures and adverse events such as suspicion of aspiration or regurgitation, hypoxia (SpO_2_ < 93%), bronchospasm, laryngospasm, dental, tongue or lip trauma were recorded.

Learning curves were established for overall time to successful intubation and for first attempt success rate.

### Study aims

This was a learning curve study for the Bonfils and the SensaScope. Advanced statistical methods to establish such learning curves were applied to provide possible guidance for future learning curve studies for procedural skills.

### Statistical analysis

Statistical analyses were performed with Stata V.15.1 (StataCorp, College Station, TX, USA) and R 3.3.0 (The R Foundation, www.r-project.org) for fitting models.

Data are presented as number and percent for binary data, or as median with interquartile range for continuous data.

To establish learning curves of each device we fitted mixed-effects regression models. Time to successful intubation was log-transformed to improve normality and fitted with linear mixed-effects regression and the first attempt success probability was fitted with logistic mixed-effects regression models using R functions lmer and glmer, respectively, from package lme4 [18]. Random effects consisted of a random intercept and slope for the anaesthetist. Crude models included device and trial number (as a continuous variable) as fixed covariates. In adjusted models, we added Mallampati score (dichotomized to I or II vs. III or IV), BMI, and the experience of the anaesthetists (consultants vs. registrars). For intubation time, results are presented as geometric mean ratio with 95% confidence intervals (CIs) based on the t-distribution with Satterthwaite’s approximation for the degrees of freedom [19] implemented in lmerTest [20]. Unsuccessful trials were excluded from the main analysis. For the modeled success probabilities, results are presented as odds ratio with 95% CIs based on a normal approximation. A probability of *p* ≤ 0.05 was considered statistically significant. Predictions from the adjusted models were calculated for consultants and registrars, marginalized over BMI and Mallampati (using R-package ggeffect) [21].

To test the results of the above models, we performed further sensitivity analyses. First, we analysed first attempt success within 60 s. Second, we included failed trials for overall intubation time, using a total of 480 s for a failed trial (120 s for each of the three failed attempts, plus 120 s as a penalty for overall failure). Third, we added the interaction of device and trial to the model.

This was an explorative study with no formal sample size calculation. The study subjects were the anaesthetists and the number of study participants was based on studies performed by Biro et al., who studied 8 anaesthetists with 4 intubations each [3] and Falcetta et al. who studied 5 anaesthetists [12]. We intended to include 15 registrars and 15 consultants, each with 10–20 intubations with the Bonfils and with the SensaScope. A recent study by Altun et al., which was unpublished when the presented study was planned, included 15 anaesthetists [14].

## Results

Fifteen consultants (median experience 11 years, IQR 10–16 years) and 15 registrars (median experience 0 years, IQR 0–1.5 years) participated. Two consultants were female and 13 were male. Seven registrars were female and 8 were male. Twenty doctors completed 10 trials, 4 completed 15 trials and 6 doctors completed 16 to 20 trials with each device.

A total of 740 trials of intubation were performed between February 72,011 and March 242,014: 370 with the SensaScope and 370 with the Bonfils. This corresponded to inclusion of 736 different patients, as 4 patients were included twice (3 were randomized to both groups once, 1 was randomized to the Bonfils group twice). As randomisation was performed just before the study intervention, all patients received the intended treatment and data of all 740 intubations were analysed. Table 1 indicates demographic data of the patients.

**Table 1:** Patient demographics

	Bonfils	SensaScope
Number^a^	369	370
Females	163 (44%)	161 (44%)
Age (years)	56.0 [43.0, 68.0]	53.0 [41.0, 66.0]
Height (cm)	170 [164, 178]	171 [165, 178]
Weight (kg)	75.0 [65.0, 85.0]	74.0 [64.0, 86.0]
BMI (kg^a^m-2)	25.5 [22.9, 28.7]	25.4 [22.4, 28.5]
ASA class I/ II/ III ^b^	74/ 173/ 121 (20/ 47/ 33%)	74/ 183/ 113 (20/ 49/ 31%)
Mallampati I/ II/ III/ IV ^c^	179/ 162/ 28/ 0 (49/ 44/ 8/ 0%)	179/ 152/ 35/ 1 (49/ 41/ 10/ 0%)

Data are number and percent, or median and interquartile range

^a^*732 patients were included once: 367 were randomised to the SensaScope group, 365 were randomised to the Bonfils group. 4 patients were included in the study twice: 3 were randomised to both groups once, 1 was randomised to the Bonfils group twice*

^b^ *Missing data for 1 patient with Bonfils*

^c^ *Missing data for 3 patients with SensaScope*

**Fig. 1:**
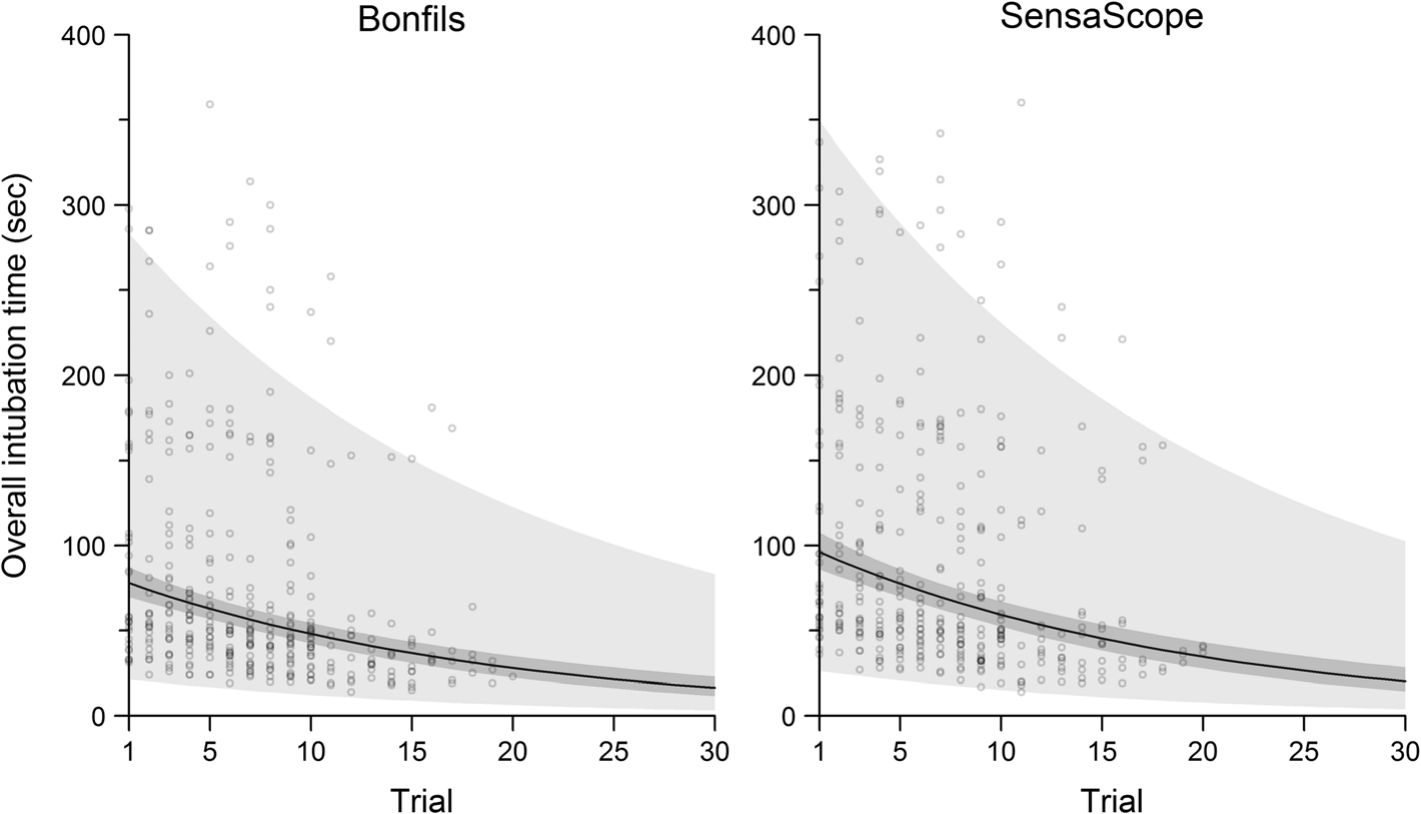
Predicted overall intubation time with 95% confidence intervals (dark grey) and 95% prediction intervals (light grey) from the linear mixed-effects regression model. The raw data is indicated with circles. Data points beyond 20 trials are out of sample predictions

Figure 1 shows the learning curve for overall intubation time from the linear mixed-effects regression model for the devices, demonstrating a decrease in the predicted overall intubation time with increasing numbers of trials.

**Fig. 2:**
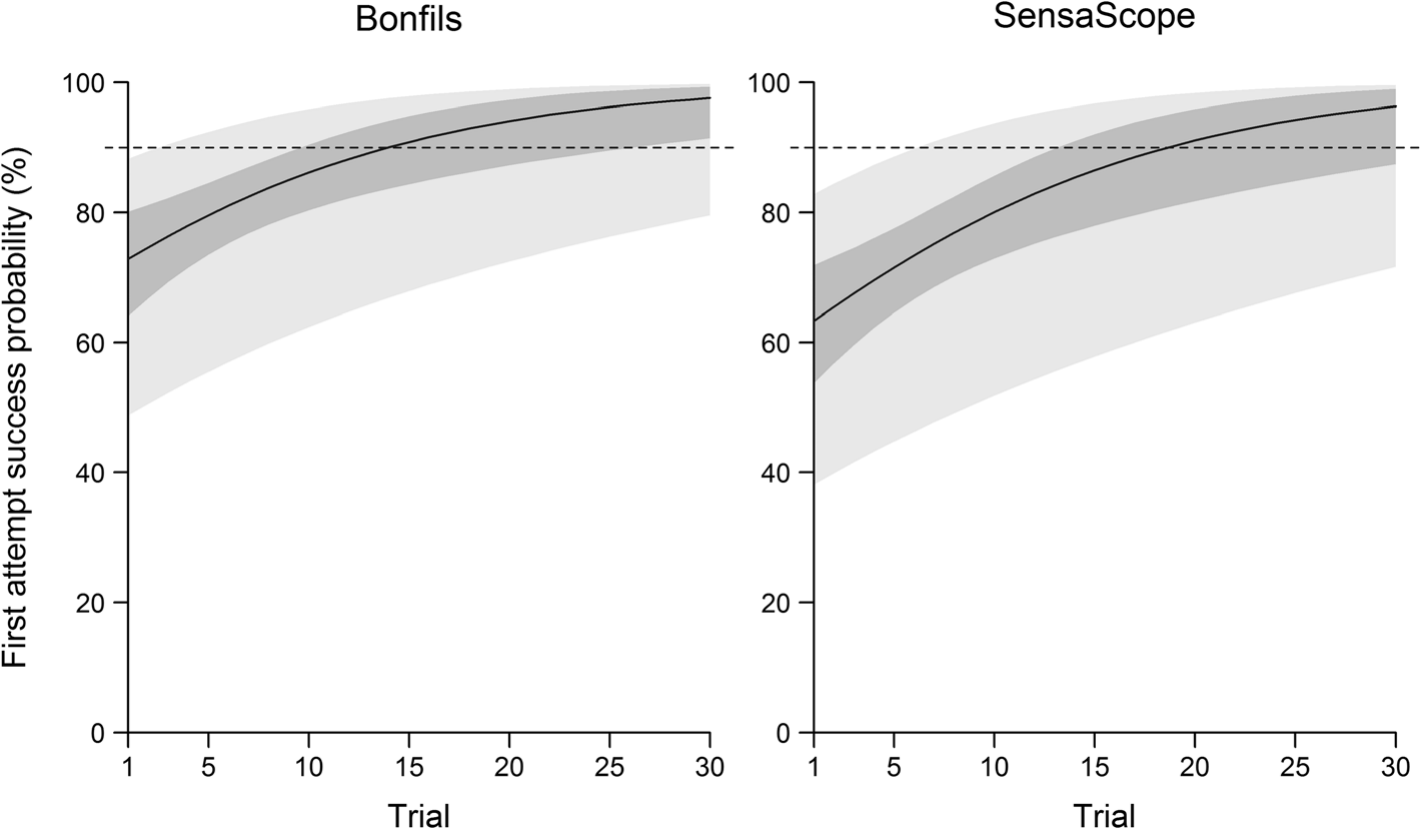
Predicted first attempt success probability with 95% confidence intervals (dark grey) and 95% prediction intervals (light grey). In order to reach a 90% first attempt success probability, 14 and 20 trials are needed with Bonfils and SensaScope, respectively

Similarly, Fig. 2 shows the learning curve for first attempt success probability from a logistic mixed-effects model for the devices, demonstrating an increase of the predicted first attempt success probability with increasing number of trials. To reach a 90% first attempt success probability in average, 14 and 20 trials are needed with the Bonfils and the SensaScope, respectively.

The improvement of intubation times and success rates can also be seen in Table 2, separately analysed for registrars and consultants. For example, intubation by registrars with the Bonfils took 79 s (95% CI 68–91 s) on the first trial and only 28 (95% CI 22–36) on the 20th trial. First attempt success probability of registrars using the Bonfils increased from 67% (95% CI 56–77%) on the first trial to 93% (95% CI 84–97%) on the 20th trial.

**Table 2:** Time to successful intubation (seconds) and first attempt success probability

	Trial	Seconds to intubation (95% CI)			First attempt success probability (95% CI)		
		Crude	Adjusted - Registrars	Adjusted - Consultants	Crude	Adjusted - Registrars	Adjusted - Consultants
Bonfils	1	78 (70–87)	79 (68–91)	77 (67–90)	73% (64–80%)	67% (56–77%)	78% (68–85%)
	5	63 (57–70)	64 (55–73)	62 (54–72)	80% (73–85%)	75% (66–82%)	84% (77–89%)
	10	48 (42–54)	49 (41–57)	48 (41–56)	86% (80–90%)	83% (75–89%)	89% (83–93%)
	15	37 (31–43)	37 (30–45)	36 (30–44)	91% (84–95%)	89% (80–94%)	93% (87–96%)
	20	28 (22–35)	28 (22–36)	28 (22–35)	94% (87–97%)	93% (84–97%)	95% (90–98%)
SensaScope	1	96 (86–108)	97 (84–113)	95 (82–110)	63% (54–72%)	57% (46–68%)	69% (58–79%)
	5	78 (70–86)	78 (68–90)	77 (67–88)	72% (65–78%)	66% (57–74%)	77% (69–83%)
	10	59 (52–67)	60 (51–70)	59 (50–69)	80% (73–86%)	76% (66–83%)	84% (77–90%)
	15	45 (38–54)	46 (37–56)	45 (37–54)	87% (78–92%)	83% (72–91%)	90% (82–94%)
	20	35 (28–43)	35 (27–45)	34 (27–44)	91% (82–96%)	89% (77–95%)	93% (85–97%)

Predicted from the crude model and from the adjusted model for registrars and consultants

The effect of device and trial number on the overall time to successful intubation and on first attempt success probability is shown in Tables 3 and 4. Both device and trial number have a statistically significant effect on the overall intubation time and on first attempt success probability. The geometric mean intubation time with the SensaScope was 1.23 times longer than with the Bonfils (95% CI 1.12–1.36, *p* < 0.001), and the odds of having success at a given trial was reduced by a factor of 0.64 with the SensaScope compared to the Bonfils (95% CI 0.45–0.92, *p* = 0.016).

**Table 3:** Effects on overall time to successful intubation (in seconds)

	Crude model		Adjusted model	
	Geometric mean ratio (95% CI)	p-value	Geometric mean ratio (95% CI)	p-value
Device (SensaScope vs. Bonfils)	1.23 (1.12–1.36)	< 0.001	1.23 (1.12–1.36)	< 0.001
Trial	0.95 (0.94–0.96)	< 0.001	0.95 (0.93–0.96)	< 0.001
Mallampati (III/IV vs I/II)			1.06 (0.90–1.26)	0.47
BMI (per unit increase)			1.00 (0.99–1.01)	0.79
Consultant vs. registrar			0.98 (0.81–1.19)	0.85
Intercept	82.2 (72.9–92.7)	< 0.001	80.1 (60.3–106.4)	< 0.001

Effects expressed as geometric mean ratio and 95% confidence intervals (CI) from a crude model with device and trial number and an adjusted model with Mallampati score, BMI, and physician (consultant vs. registrar)

**Table 4:** Effects on first attempt success

	Crude model		Adjusted model	
	Odds ratio (95% CI)	*p*-value	Odds ratio (95% CI)	p-value
Device (SensaScope vs Bonfils)	0.64 (0.45–0.92)	0.016	0.65 (0.46–0.93)	0.020
Trial	1.10 (1.04–1.16)	< 0.001	1.10 (1.04–1.16)	< 0.001
Mallampati (III/IV vs. I/II)			0.88 (0.47–1.63)	0.68
BMI (per unit increase)			0.97 (0.94–1.01)	0.15
Consultant vs. registrar			1.70 (1.00–2.88)	0.05
Intercept	2.44 (1.57–3.78)	< 0.001	3.67 (1.34–10.07)	0.011

Effects expressed as odds ratio with 95% confidence intervals (CI) from a crude model with device and trial number and an adjusted model with Mallampati score, BMI, and physician (consultant vs. registrar)

We included the potentially relevant covariables Mallampati, BMI and physician (consultant vs. registrar) in both models. We did not find any evidence that Mallampati, BMI or physician would influence the intubation time (*p* = 0.47, *p* = 0.79, *p* = 0.85) nor did we find evidence that Mallampati or BMI would influence first attempt success (*p* = 0.68 and *p* = 0.15). We only found a tendency that consultants may increase the odds for first attempt success compared to registrars (odds ratio 1.7, 95% CI 1.00–2.88, *p* = 0.05).

### Sensitivity analysis

Restricting first attempt success to a success within 60 s did not have a major effect on the results: The odds ratio for first attempt success for Bonfils vs. SensaScope decreased from 0.64 (95% CI 0.45–0.92) to 0.55 (0.39–0.77).

The modelled learning curve did not change when overall failed intubations were included and counted as 480 s. The geometric mean ratio for overall intubation time of the SensaScope vs. the Bonfils was 1.24 (95% CI 1.12–1.38) which is very similar to the main analysis.

Finally, we did not find evidence that the effect of the device would change over the trials, i.e. the interaction of device and trial was not significant (*p* = 0.54 for overall intubation time and *p* = 0.76 for first attempt success probability).

### Failures and adverse events

Twenty-three of the 740 intubations failed (10 Bonfils, 13 SensaScope). Reasons for intubation failures are given in Table 5. Adverse events were rare and are also given in Table 5.

**Table 5:** Failures and adverse events. Data are number (percent)

	Bonfils *n* = 370	SensaScope n = 370
**Intubation failures**	**10 (3%)**	**13 (4%)**
Lack of visualisation of glottis	5 (1%)	6 (2%)
Oesophageal intubation	2 (1%)	1 (0%)
Excessive salivation	2 (1%)	0 (0%)
Bleeding	0 (0%)	1 (0%)
Failure to advance tube	0 (0%)	1 (0%)
Time limit (3 × 360 s)	1 (0%)	4 (1%)
Minor lip trauma	1 (0%)	4 (1%)
Mucosal bleeding	0 (0%)	1 (0%)
Dental trauma	0 (0%)	1 (0%)

There was no tongue trauma, aspiration or regurgitation, hypoxia, bronchospasm or laryngospasm.

## Discussion

This study established learning curves for the rigid scopes Bonfils and SensaScope used by 30 anaesthetists for 740 elective intubations. A clear learning process was demonstrated for both devices: First attempt intubation success rates increased by 21 to 28 percentage points (Bonfils vs. SensaScope) and intubation time improved roughly 2.5-fold over the first 20 trials of intubation, leading to an intubation time of 28 to 35 s. These numbers show that after the initial learning process, intubation with the rigid scopes can be carried out quickly in a timeframe which most patients will easily tolerate without desaturations [22]. Indeed, none of the 740 patients desaturated below 93%.

The overall intubation time with the SensaScope was 1.23 times longer than the overall intubation time with the Bonfils (geometric mean ratio 1.23 (95% CI 1.12–1.36, *p* < 0.001). Intubation time was dependent on the device used and on the experience of the anaesthetist with the device (trial number), but it did not depend on other variables such as Mallampati class, BMI or the overall clinical experience (years on the job) of the doctor. Using the data of the presented model, it is possible to predict overall intubation time at a given trial (compare Table 3 and Fig. 1). Overall intubation time can be predicted as the intercept multiplied by the geometric mean ratio of the device (1 for Bonfils, 1.23 for SensaScope) multiplied by the geometric mean ratio of the trial. For example, the predicted intubation time with the Bonfils on the first trial is 82.2*1*0.9477 = 78 s, while the predicted intubation time with the SensaScope on the 10th trial is 82.2*(0.9477^10)*1.23 = 59 s.

To reach a 90% success probability, 14 intubations are necessary with the Bonfils and 20 with the SensaScope. This is substantially more than was described by Biro et al. who described a flattening of the learning curve with the SensaScope after only two intubations [3]. However, Biro’s study comprised a total of only 32 intubations, performed by 8 operators each intubating 4 times, and used purely graphical methods to assess a learning curve. Studies regarding the Bonfils suggested figures similar to our results, estimating that 10 to 20 intubations are necessary to achieve proficiency [12, 13]. Interestingly, these figures all support the notion that it might be faster to learn intubation with rigid scopes than to learn intubation with the Macintosh laryngoscope with a recommended caseload of 57 intubations [8]. An alternative explanation might be that anaesthetists intubating with rigid scopes all had prior experience with conventional intubation. It is possible that expertise with one skill might enhance the learning of a related skill (“transfer effect”) [23]. Also, the higher first attempt success rate of consultants only just missed statistical significance (*p* = 0.05, Table 4). Again, this supports the notion that a transfer effect from one skill to another might be present and consultants trained on flexible optical scopes may benefit from these acquired skills.

With regards to the device performance: Significant differences were found in first attempt success rates (OR 0.64, 95% CI 0.45–0.92, Table 4), and overall time to intubation (geometric mean ratio 1.23, 95% CI 1.12–1.36, Table 3). All these differences showed a better performance of the Bonfils compared to the SensaScope. These analyses were supported by several sensitivity analyses, which all showed superiority of the Bonfils over the SensaScope. However, this difference is less pronounced and clinically irrelevant after the early learning phase (Table 2 and Fig. 2). It is possible that the somewhat prolonged learning curve of the SensaScope is due to an increased complexity of the device which does not feature a completely rigid stylet, but a steerable tip in addition. It will be interesting to compare these findings with the performance of the recently introduced C-MAC Videoscope (Karl Storz, GmbH, Tuttlingen, Germany), which is a Bonfils-shaped scope with a steerable tip similar to the SensaScope.

When looking at the reasons for failure, there was no difference between the devices. Most often, the glottic inlet could not be identified (48%). Contrary to the situation with videolaryngoscopes, problems with advancing the tube were rarely encountered. It seems that in contrast to videolaryngoscopes the “you see that you fail” situation, where the glottis is identified, but the trachea cannot be intubated [24], is not a problem with rigid scopes. Also, adverse events were rare. Given the high success rates of intubation after the initial learning of the technique [1], rigid scopes might be a valuable alternative for videolaryngoscopes in (difficult) airway management.

### Limitations

With no data points beyond 20 trials, the presented learning curves beyond the 20th intubation are out of sample predictions and have to be interpreted with care. However, we included 30 anaesthetists, which is more than any trial on learning curves of rigid scopes before, and our sensitivity analyses support the validity of the presented learning curves. Learning will always be an individual process, but we believe that the present study, with its large number of anaesthetists and intubations, represents a good picture of learning curves for the two studied devices.

## Conclusions

A clear learning effect was demonstrated for both rigid scopes. Intubation times decreased roughly 2.5-fold and first attempt intubation success probability increased by 21–28 percentage points. Fourteen intubations with the Bonfils and 20 intubations with the SensaScope were required to reach a 90% first attempt success probability. Performance was overall slightly better with the Bonfils, particularly during the early learning phase. Success rates with both devices were high after the initial learning phase and adverse events were rare, indicating that both devices could serve as valuable airway tools in experienced hands.

## Data Availability

The datasets used and analysed during the current study are available from the corresponding author on reasonable request.

## Abbreviations

ASA: American Society of Anesthesiologists
BMI: Body mass index
CI: Confidence interval
IQR: Interquartile range
OR: Odds ratio
